# Nationwide Analysis of Food Insecurity among Individuals with Inflammatory Bowel Disease in the United States

**DOI:** 10.1093/ibd/izaf289

**Published:** 2025-11-19

**Authors:** Mark R Baniqued, Alexandra C Greb, Neha D Shah, Alyssa A Parian, Zhaoping Li, Berkeley N Limketkai

**Affiliations:** Department of Medicine, UCLA-Olive View Medical Center, Los Angeles, CA, United States; Department of Medicine, UCLA Ronald Reagan Medical Center, Los Angeles, CA, United States; Colitis and Crohn’s Disease Center, University of California, San Francisco, CA, United States; Department of Gastroenterology, Hackensack University Medical Center, Hackensack, NJ, United States; Division of Clinical Nutrition, UCLA School of Medicine, Los Angeles, CA, United States; Division of Clinical Nutrition, UCLA School of Medicine, Los Angeles, CA, United States; Vatche and Tamar Manoukian Division of Digestive Diseases, UCLA School of Medicine, Los Angeles, CA, United States

**Keywords:** diet, disparities, equity, Supplemental Nutrition Assistance Program

## Abstract

**Background:**

Diet impacts symptoms and inflammation in inflammatory bowel disease (IBD), but limited food access restricts options and hampers adherence to diets that may help in the management of IBD. This study aims to measure the prevalence of food insecurity and characterize its risk factors in the adult population across the United States.

**Methods:**

The 2023, 2016, and 2015 National Health Interview Surveys were queried for individuals with and without IBD. Food insecurity prevalence and sociodemographic characteristics were compared. Multivariable logistic regression identified factors associated with food insecurity risk, while considering the survey’s complex sampling design.

**Results:**

In 2023, a significantly higher proportion of individuals with IBD (*n* = 4 289 979) reported food insecurity compared to those without IBD (*n* = 253 490 081) (13.48% vs 8.96%, *P* = 0.01). The pooled 2015–2016 data showed similar findings (12.43% vs 9.43%, *P* < 0.01). No significant difference was found across these periods (*P* = 0.63). Multivariable models linked higher food insecurity risk with IBD status (OR 1.85, *P* < 0.01), female sex (OR 1.16, *P* < 0.01), Hispanic ethnicity (OR 1.24, *P* < 0.01), non-Hispanic Black race (OR 1.88, *P* < 0.01), having Medicaid (OR 1.85, *P* < 0.01), being uninsured (OR 2.02, *P* < 0.01), and use of Supplemental Nutrition Assistance Program (SNAP) (OR 2.49, *P* < 0.01).

**Conclusions:**

Individuals with IBD face a persistently higher risk of food insecurity. Some of the most important predictors included being non-Hispanic Black, being uninsured or having Medicaid insurance, and using SNAP. Clinicians should regularly screen patients with IBD for food insecurity and address it through a multidisciplinary approach, involving dietitians and social workers to enhance patient care and outcomes.

Key Messages:

- What is already known?

Diet is key in managing symptoms and inflammation in inflammatory bowel disease, but little research exists on the prevalence and impact of food insecurity in this population.

- What is new here?

This study utilizes the 2023 National Health Interview Survey, which confirms that food insecurity is prevalent and persistent among patients with inflammatory bowel disease and identifies additional social and demographic risk factors for food insecurity.

- How can this study help patient care?

This study emphasizes the importance of screening for and addressing food insecurity, especially in patients with inflammatory bowel disease, and recommends ways to do so.

## Introduction

Patients with inflammatory bowel disease (IBD) often have diverse beliefs about the role of diet in managing their condition.^1^ Food aversion and avoidant behavior are common in patients with IBD, regardless of the severity of their disease.^2^ Based on their experiences, they may identify specific foods that exacerbate their symptoms and heighten their susceptibility to flares. These patients are already at a greater risk of nutritional deficiencies due to the chronic inflammation linked to their disease process.^3^ In addition to their disease, these individuals may also face social factors such as food insecurity (FI), which can further limit their food options.

The United States Department of Agriculture (USDA) defines FI as “the limited or uncertain availability of nutritionally adequate and safe foods or limited or uncertain ability to acquire acceptable foods in socially acceptable ways.”^4^ Food security status exists on a spectrum of 4 categories: high, marginal, low, and very low food security. FI is defined as low or very low food security. FI is a global issue, even in high-income countries like the United States, where it often remains hidden, and the common perception is that it is not a significant concern.^5^ Individuals with FI face an increased risk of diet-related diseases such as diabetes, hypertension, cardiac disease, increased emergency room visits, and hospitalizations.^6^

The effect of FI on chronic disease pathogenesis and management has become a growing area of interest.^7^ However, data on the impact of FI in IBD are scarce. In a narrative review published in 2023, only 12 studies had examined FI in digestive diseases, with only one involving IBD.^7^ Nguyen et al assessed the associations of financial toxicity with clinical outcomes in the 2015 National Health Interview Survey (NHIS).^8^ They found that 14% of patients with IBD suffer from FI and lack social support. Patients facing FI were found to experience higher rates of cost-related medication non-adherence. A single-center study published by Gold et al in 2024 noted that FI was prevalent in the 128 patients with IBD, and patients at high risk for FI were significantly more likely to eat ultra-processed foods (54% vs 27%).^9^ They also noted that only 13% of patients with the highest risk for FI were enrolled in a federal food assistance program. In a survey study by Kim et al., they found multiple social risks among their adult IBD patient population, with FI (22%) and financial hardship (28%) being some of the most prevalent.^10^ This higher burden of social risk was associated with increased risk of unplanned healthcare utilization and issues with medication adherence related to cost.

Overall, FI restricts the food options for individuals with IBD and may worsen their symptoms, negatively impacting their overall health. FI would also intensify the risk of malnutrition by impacting the quality of patients’ diet and restricting their ability to utilize dietary interventions. Using a nationally representative dataset, this study aims to examine the prevalence of FI among individuals with and without IBD and to identify its risk factors within the adult population across the United States. We hypothesize that individuals with IBD have an increased risk for FI and that socioeconomic factors contribute to this risk.

## Methods

The 2023 NHIS was queried for individuals with and without IBD. The NHIS, a cross-sectional household survey carried out by the US Centers for Disease Control and Prevention, tracks the health of civilians throughout the United States. Annually, information is gathered from approximately 27 000 randomly chosen adults representing their households via face-to-face interviews.^11^ The survey evaluates health topics, including various health conditions, health behaviors, access and utilization of healthcare, and demographic factors. Prior to 2023, the last inclusion of IBD data in the NHIS occurred in 2016. In 2023, patients were identified as being diagnosed with IBD based on a “yes” response to the survey question: “Have you EVER been told by a doctor or other health professional that you had Crohn’s disease?” and “Have you EVER been told by a doctor or other health professional that you had Ulcerative Colitis?” Individuals were categorized into 4 food security levels: high, marginal, low, and very low. Individuals were coded into various categories based on their responses to the USDA Food survey questionnaire included in the NHIS to determine the overall FI prevalence. The overall FI prevalence included the components of FI such as “Worry food would run out,” “Food didn’t last,” “Couldn’t afford to eat balanced meals,” “Cut the size of meals or skip meals,” “Eat less than should,” “Ever hungry because not enough money for food,” “Lose weight because not enough money for food,” and “Not eat for a whole day.” Those experiencing FI are classified as low or very low food security. Supplemental Nutrition Assistance Program (SNAP) use was coded as binary, either “yes” or “No/Refused/Don’t Know” responses. Similarly to querying the 2023 NHIS, the 2015 and 2016 data were also queried for the same food security and IBD variables. Data from 2015 and 2016 were pooled together as both years were close temporally and were not expected to derive any meaningful differences between these 2 time points. These 2 datasets used the same methodology and variable definitions.

### Potential Factors

Data were also collected on the following potential contributors to FI: age, sex (male/female), race/ethnicity (non-Hispanic White, Hispanic, non-Hispanic black, non-Hispanic Asian, non-Hispanic American Indian Alaskan Native), health insurance (private, Medicare only, Medicaid, other, uninsured), food stamps (yes/no/refused), and the ratio of family income to poverty level. Other health insurance grouped the following NHIS survey responses: Medigap, Military-Related, Indian Health, State-Sponsored, and Other Government Program.

### Statistical Analyses

The prevalence of FI and sociodemographic characteristics were compared between adults with and without IBD. Categorical variables were compared using the chi-squared test with Rao-Scott modification. Continuous variables were compared using the Student *t*-test. Multivariable logistic regression evaluated factors associated with the risk for FI while adjusting for potential confounders. Statistical significance was defined as an alpha threshold of 0.05. All analyses were conducted with R 4.3 (Indianapolis, IN) and accounted for the complex sampling design of the NHIS.

## Results

The study included 253 490 081 adult individuals, of whom an estimated 4 289 979 reported having IBD in 2023. Compared with the general population, those with IBD had a higher mean age (55.8 vs 47.9 years; *P* < 0.01) (Table 1). The majority of those with IBD were females (57.2%) and non-Hispanic White (76.4%). Most individuals with IBD had private insurance (62.0%). Comparing those with and without IBD, there was a similar distribution of individuals on SNAP (15.1% vs 12.8%) and those reporting a family income-to-poverty ratio of 1 or less (7.9% vs 7.0%).

**Table 1.** Demographic data in non-IBD and IBD.

Variables	Prevalence		
	Non-IBD, % (*n* = 253 490 081)	IBD, % (*n* = 4 289 979)	*P*
**Age**			<.01
** 18-19**	3.36	0.34	
** 20-29**	17.13	7.30	
** 30-39**	17.46	15.64	
** 40-49**	15.92	12.49	
** 50-59**	15.64	18.19	
** 60-69**	15.30	18.86	
** 70-79**	10.24	17.78	
** 80-84**	2.68	4.47	
** 85+**	2.27	4.93	
**Sex**			.12
** Male**	48.92	42.81	
** Female**	51.08	57.19	
**Race/ethnicity**			<.01
** Hispanic**	17.62	12.26	
** Non-Hispanic White**	61.66	76.40	
** Non-Hispanic Black**	11.89	6.34	
** Non-Hispanic Asian**	6.35	2.27	
** Non-Hispanic American Indian and Alaska Native**	0.60	0.73	
** Other single/multiple**	1.88	1.99	
**Health insurance**			<.01
** Private**	62.97	62.03	
** Medicare only**	10.81	15.60	
** Medicaid**	12.65	10.47	
** Other**	5.01	7.43	
** Uninsured**	8.57	4.46	
**Region**			.86
** Northeast**	17.47	15.82	
** Midwest**	20.59	20.68	
** South**	38.34	38.92	
** West**	23.59	24.58	
**Supplemental Nutrition Assistance Program**			.22
** Yes**	12.75	15.06	
** No**	86.93	84.83	
** Refused**	0.32	0.11	
**Ratio of family income to poverty level**			.75
** 0 to 1**	7.03	7.87	
** 1 to 2**	20.99	21.66	
** 2+**	71.98	70.47	

Prevalence of IBD among various demographic variables compared to individuals without IBD. Abbreviation: IBD, inflammatory bowel disease.

**Figure 1.**
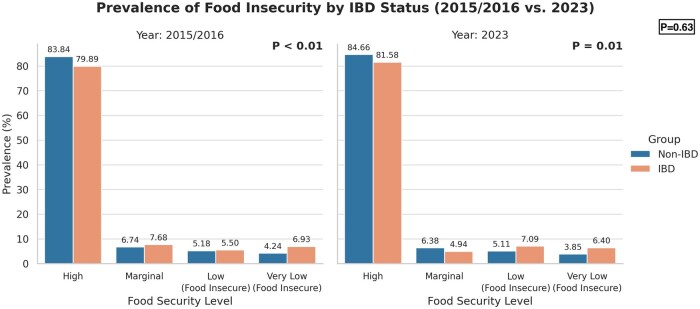
Prevalence of food insecurity by IBD status in 2015–2016 versus 2023.

Significantly more individuals with IBD reported having FI overall compared to those without IBD (13.48% vs 8.96%, *P* = 0.01) (Table 2). The pooled 2015–2016 NHIS results, which had an average non-IBD population of 240 345 706 and an IBD population of 3 121 387, similarly showed that significantly more individuals with IBD reported having FI compared to those without IBD (12.43% vs 9.43%, *P* < 0.01) (Figure 1). When comparing the prevalence of FI among individuals with IBD from 2015–2016 to 2023, there was no significant difference (*P* = 0.63). There was also no significant difference in FI among individuals without IBD (*P* = 0.13) from these 2 points in time. Among those with IBD and FI in 2023, only 46.9% were reported to be enrolled in SNAP.

**Table 2.** Food insecurity in non-IBD and IBD.

Variables	Prevalence		
	Non-IBD, % (*n* = 253 490 081)	IBD, % (*n* = 4 289 979)	*P*
**Food security levels**			.01
** High**	84.66	81.58	
** Marginal**	6.38	4.94	
** Low (Food Insecure)**	5.11	7.09	
** Very low (Food Insecure)**	3.85	6.40	
**Components of food security**			
**“Worry food would run out”**			.07
** Often**	3.97	6.31	
** Sometimes**	8.40	8.52	
** Never**	87.63	85.18	
**“Food didn’t last”**			<.01
** Often**	3.13	6.84	
** Sometimes**	7.59	6.90	
** Never**	89.28	86.26	
**“Couldn’t afford to eat balanced meals”**			<.01
** Often**	3.22	6.92	
** Sometimes**	7.02	8.53	
** Never**	89.76	84.55	
**“…did you/adults in the family cut the size of meals or skip meals”**			.1
** Yes**	35.78	45.16	
** No**	64.22	54.84	
**“Eat less than should”**			.03
** Yes**	35.75	47.88	
** No**	64.25	52.12	
**“Ever hungry because not enough money for food”**			.04
** Yes**	22.83	33.08	
** No**	77.17	66.92	
**“Lose weight because not enough money for food”**			<.01
** Yes**	14.55	25.84	
** No**	85.45	74.16	
**“Not eat for a whole day”**			.27
** Yes**	18.93	25.77	
** No**	81.07	74.23	

Statistical analysis of the comparison of coded food security at 4 levels based on responses to component questions between US adults with and without IBD. Food insecurity is defined as when an individual has low or very low food security. Abbreviation: IBD, inflammatory bowel disease.

The FI components with significant disparities in 2023 included: “Food didn’t last” (*P* < 0.01), “Couldn’t afford to eat balanced meals” (*P* < 0.01), “Eat less than should” (*P* = 0.03), “Ever hungry because not enough money for food” (*P* = 0.04), and “Lose weight because not enough money for food” (*P* = 0.02). Components without a significant difference between individuals with IBD compared to those without were: “Worry food would run out,” “Cut the size of meals or skip meals,” or “Not eat for a whole day.” The key FI components with significant disparities in 2015–2016 were as follows: “Worry food would run out” (*P* = 0.01), “Food didn’t last” (*P* = 0.02), “Cut the size of meals or skip meals” (*P* = 0.01), “Every hungry because not enough money for food” (*P* < 0.01), and “Lose weight because not enough money for food” (*P* = 0.03).

In the multivariable models, higher FI risk was associated with IBD (odds ratio [OR] 1.85, 95% CI 1.33-2.55, *P* < 0.01), Age 50-60 (OR 1.54, 95% CI 1.03-2.30, *P* = 0.04), Female sex (OR 1.16, 95% CI 1.04-1.29, *P* < 0.01), Hispanic race (OR 1.24, 95% CI 1.06-1.46, *P* < 0.01), non-Hispanic Black race (OR 1.88, 95% 1.60-2.21, *P* < 0.01), Other single/multiple races (OR 1.64, 95% CI 1.12-2.40, *P* = 0.01), having Medicaid insurance (OR 1.85, 95% CI 1.56-2.19, *P* < 0.01), other insurance (OR 1.76, 95% CI 1.41-2.29, *P* < 0.01), being uninsured (OR 2.02, 95% CI 1.67-2.45, *P* < 0.01), and SNAP use (OR 2.49, 95% CI 2.16-2.88, *P* < 0.01) (Table 3). Conversely, lower FI risk was associated with Age 85+ (OR 0.47, 95% CI 0.25-0.86, *P* = 0.01), non-Hispanic Asian ethnicity (OR 0.52, 95% CI 0.38–0.71, *P* < 0.01), and having a higher income-to-poverty ratio (OR 0.47, 95% CI 0.43-0.51, *P* < 0.01). Multicollinearity was tested and not found to be present. The adjusted generalized variance inflation factors of the primary model ranged from 1.0 to 1.3 among covariates.

**Table 3.** Multivariable logistic regression of food insecurity outcome.

Variables		Outcome = food insecurity	
		OR (95% CI)	*P*
**IBD**		1.85 (1.33-2.55)	<.01
**Age**	18 to 20	Reference	
	20 to 30	1.29 (0.86-1.96)	.22
	30 to 40	1.47 (0.98-2.19)	.06
	40 to 50	1.47 (1.00-2.17)	.05
	50 to 60	1.54 (1.03-2.30)	.04
	60 to 70	1.13 (0.75-1.71)	.56
	70 to 80	0.97 (0.63-1.50)	.89
	80 to 85	0.61 (0.36-1.05)	.07
	85+	0.47 (0.25-0.86)	.01
**Sex**	Male	Reference	
	Female	1.16 (1.04-1.29)	<.01
**Race/ethnicity**	Non-Hispanic White	Reference	
	Hispanic	1.24 (1.06-1.46)	<.01
	Non-Hispanic Black	1.88 (1.60-2.21)	<.01
	Non-Hispanic Asian	0.52(0.38-0.71)	<.01
	Non-Hispanic American Indian and Alaska Native	1.25 (0.81-1.93)	.30
	Other single/multiple	1.64 (1.12-2.40)	.01
**Health insurance**	Private	Reference	
	Medicare only	1.18 (0.97-1.45)	.11
	Medicaid	1.85 (1.56-2.19)	<.01
	Other	1.76 (1.41-2.20)	<.01
	Uninsured	2.02 (1.67-2.45)	<.01
**Supplemental Nutrition Assistance Program**	No	Reference	
	Yes	2.49 (2.16-2.88)	<.01
**Ratio of family income to poverty level**		0.47 (0.43-0.51)	<.01

Multivariable logistical regression analysis for food insecurity outcome when adjusted for IBD, age, sex, race/ethnicity, health insurance, Supplemental Nutrition Assistance Program use, and ratio of family income to poverty level. Abbreviation: IBD, inflammatory bowel disease.

A subgroup analysis of FI was also conducted to examine the relationship between race/ethnicity and IBD status. This was conducted for both the 2023 and the pooled 2015–2016 datasets. This indicated no significant difference among different race/ethnicity categories in the IBD group in both 2023 (*P* = 0.067) and 2015–2016 (*P* = 0.016) (Table 4). There was a significant difference noted between these categories in the non-IBD group in both 2023 (*P* < 0.001) and 2015–2016 (*P* < 0.001).

**Table 4.** Sub-group analysis of food insecurity between race/ethnicity and IBD.

	2023					
	No IBD			IBD		
Race/ethnicity	Food secure, %	Food insecure, %	*P* < .001	Food secure, %	Food insecure, %	*P* = .067
**Hispanic**	86.46	13.54		80.30	48.15	
**NH White**	93.48	6.52		88.83	48.72	
**NH Black**	82.73	17.27		72.22	46.61	
**NH Asian**	96.67	3.33		91.15	48.53	
**NH AIAN**	82.29	17.71		85.13	50.85	
**Other single/multiple**	87.62	12.38		73.46	45.61	

	**2015**–**2016**					
	No IBD			IBD		
Race/ethnicity	Food secure, %	Food insecure	*P* < 0.001	Food secure, %	Food insecure, %	*P* = .016
**Hispanic**	85.61	14.39		83.91	16.09	
**NH White**	93.19	6.81		88.69	11.31	
**NH Black**	81.84	18.16		74.20	25.80	
**NH Asian**	96.27	3.73		98.46	1.54	
**NH AIAN**	79.25	20.75		92.08	7.92	
**Other single/multiple**	83.01	16.99		92.07	7.93	

Sub-group analysis of FI between the race/ethnicity variables and IBD. The race/ethnicity categories are Hispanic, non-Hispanic (NH) White, NH Black, NH Asian, American Indian and Alaska Native (AIAN), and other single/multiple. Abbreviation: IBD, inflammatory bowel disease.

## Discussion

In our nationwide analysis, individuals with IBD face a higher risk of FI than the average US adult. This significant risk of FI persists even when controlling for factors such as age, sex, race/ethnicity, type of health insurance, SNAP usage, and income ratio. This is particularly relevant for issues like food scarcity, the expense of balanced meals, under-eating, and weight loss due to insufficient funds. Additional risk factors for FI include being Age 50-60, female, Hispanic, non-Hispanic Black, having Medicaid insurance, utilizing SNAP, or being uninsured. Individuals over the age of 85, non-Hispanic Asian, or with higher income levels were at a reduced risk for FI. We also note that there has not been a significant change in FI among patients with IBD in the latest results from 2023 compared to 2015–2016. This problem has persisted over the last 8 years, leaving an unrealized opportunity for public health advancement.

Individuals with IBD and reduced access to adequate food are at risk for nutritional deficiencies and exacerbation of GI symptoms, which can worsen the patient’s condition overall. This is especially important for individuals with IBD since diet significantly affects bowel inflammation and related symptoms. Individuals with IBD are already known to have a higher risk for malnutrition, and FI would only increase this risk factor. Additionally, various studies have demonstrated the benefits of potential dietary interventions in managing IBD.^12^ These individuals would not be able to utilize these interventions to manage their symptoms and inflammation. Poor access to food has also been associated with lower medication adherence overall. A meta-analysis studying the effects of social determinants of health (SDOH) on medication adherence found a significant relationship between FI and lower medication adherence (adjusted OR 0.56, 95% CI 0.42-0.7).^13^ FI may contribute to medication adherence challenges because individuals with FI often have limited income and must choose between paying for food or medications. FI can directly cause nutritional deficiencies because those with restricted access to food might not be able to afford or obtain nutritious foods. All of these factors are closely linked to FI, and when they negatively affect the patient’s symptoms, they can lead to increased healthcare utilization and costs. While FI negatively impacts all chronic diseases, the close relationship between nutrition, gut inflammation, and medication absorption in IBD makes FI a crucial factor in outcomes for these patients, highlighting the importance of screening for FI in this population.

Multiple studies indicate that there is already a high prevalence of financial distress among patients with IBD.^14^^,^^15^ Patients with IBD are associated with higher odds (OR 1.56, 95% CI 1.21-2.02, *P* < 0.01) of financial toxicity compared to those without IBD.^14^ It is estimated that about 1 in 4 adults with IBD experience financial hardship due to issues with medical bills, and 1 in 6 adults reports issues with medical adherence secondary to cost. Another study found that FI was strongly associated with a worse health-related quality of life for all patients.^16^ These stresses in life can often create a cycle and, when chronic, can worsen symptoms of IBD.

Demographic risk factors for higher FI risk are linked to being female, Hispanic, non-Hispanic Black, on Medicaid, or uninsured. The literature on patients with IBD that corroborates this finding is limited. The only study noted was by Damas et al, reporting a similar finding that Hispanics and non-Hispanic Blacks in the United States suffer from disproportionately higher rates of social barriers that affect their IBD management.^17^ In chronic disease in general, a study by Bittoni et al shows that patients with public health insurance are associated with a higher risk of all-cause mortality in patients with public insurance/no insurance compared to private insurance (HR 1.54, 95% CI 1.19-1.70).^18^

Unlike in this study, where various SDOH variables are treated as independent, the reality is that socioeconomic factors are often interconnected, making it difficult to link them individually to FI. An individual’s socioeconomic status can significantly influence their access to basic resources like food and housing. We acknowledge that insurance status, SNAP use, and income-to-poverty ratio could all lie along the causal pathway between IBD and FI. Empirical analyses suggest these variables are more likely to act as confounders. When sensitivity analyses were performed, excluding each covariate, the association between IBD and FI remained stable (adjusted OR 1.8–1.9), supporting their inclusion in our multivariable models. Additionally, the subgroup analysis of FI between race/ethnicity and IBD revealed no significant difference in FI prevalence across these categories within the IBD group. Furthermore, research on demographic factors and their overall impact on the disease remains limited, emphasizing the need for further investigation into how various socioeconomic factors influence individual health.

Our study revealed that protective factors included being 85 years or older, being of Asian descent, and having a higher income-to-poverty ratio. Other studies reflecting this are limited in the literature; however, SNAP has been shown to improve outcomes for patients experiencing severe FI.^19^ SNAP usage among our IBD population with FI was only 46.9%, indicating that SNAP is an underutilized resource. Nearly half of individuals with IBD and FI are not using a resource that could help reduce their financial burden. This gap may be due to a lack of awareness about available government resources or insufficient follow-up with medical providers who could recommend these programs. Resources and government food assistance programs are impactful in reducing FI, and when utilized, may assist in improving the overall health of patients with FI.

FI has important implications in IBD. These findings should encourage clinicians to screen patients with IBD for FI, which can be quickly and easily assessed using validated tools such as the USDA’s 6-item short form food security survey module or the Hunger Vital Sign tool. These assessments take just a few minutes and can offer valuable information about an individual’s access to food. If FI concerns arise, interventions such as food stamp programs and other resources could reduce FI risk and improve adherence to therapeutic diets. A multidisciplinary approach, involving referrals to dietitians and social workers, should also be adopted to address these issues.

This study’s key strength is its use of the NHIS, a large, nationally representative dataset, which ensures generalizability among individuals with IBD from diverse demographic backgrounds. The sizeable sample increases statistical power and accurate subgroup analyses. The NHIS utilizes validated survey instruments, enhancing comparability to previous studies. However, there are limitations: the NHIS data are cross-sectional, so causality between FI and demographic factors cannot be inferred. Self-reported data introduce risks of recall bias and misclassification. Due to the self-reported nature of the NHIS without confirmation of diagnosis by a gastroenterologist, the estimated IBD population in our study of 4.28 million was likely larger than the true prevalence of IBD. Furthermore, it does not differentiate IBD subtypes or report disease severity. The NHIS also excludes the institutionalized population, potentially missing groups with higher rates of FI.

In conclusion, FI is more common among individuals with IBD compared to average US adults and has been a persistent issue, with individuals with IBD having nearly twice the odds of experiencing FI. When other factors are adjusted for, individuals with IBD have a significantly increased risk for FI. A closer assessment should be considered for individuals who may be at a higher risk for FI, such as active SNAP use, and patients with public insurance or no health insurance. While causality cannot be inferred from this study’s cross-sectional design, consideration should be given to incorporating these brief screening tools into daily clinical practice. Clinicians should consider screening all patients for FI and referring them to dietitians, social workers, and food resources (eg, SNAP) using screening tools like the USDA’s 6-item food security module. Further studies, such as prospective screening studies across diverse clinical settings, should be conducted to better understand outcomes and opportunities for interventions for FI. These studies would help validate the recommendation to screen patients for FI. Additional research could investigate the characteristics and mechanisms underlying these associations, enabling the development of potential interventions and informed public policy adaptations.
